# Physiological and biochemical responses of *Isatis indigotica* to deficit irrigation in a cold and arid environment

**DOI:** 10.3389/fpls.2022.1094158

**Published:** 2023-01-12

**Authors:** Chenli Zhou, Shouchao Yu, Hengjia Zhang, Fuqiang Li

**Affiliations:** ^1^ College of Agronomy and Agricultural Engineering, Liaocheng University, Liaocheng, China; ^2^ College of Water Conservation and Hydropower Engineering, Gansu Agricultural University, Lanzhou, China

**Keywords:** antioxidant enzyme activities, malonaldehyde, photosynthetic parameter, water deficit, woad, yield, water use efficiency, proline

## Abstract

Water shortage and wastage are critical challenges to sustainable agricultural development, especially in arid and semiarid regions worldwide. *Isatis indigotica* (woad), as a traditional Chinese herb, was planted in a large area in a cold and arid environment of Hexi. Regulated deficit irrigation can reduce the growth of some vegetative organs by changing the accumulation and distribution of photosynthetic products in crops, thus increasing the economic yield of crops. In agricultural production, crop productivity may be improved by mulched drip irrigation and deficit irrigation. Hence, a field experiment was conducted to investigate the responses of photosynthesis, malondialdehyde, osmotic regulators, antioxidant enzyme activities, and the yield of woad to water deficit at different growth stages. The growth stage of woad was divided in four stages: seedling, vegetative growth, fleshy root growth, and fleshy root maturity. During vegetative growth, fleshy root growth, and fleshy root maturity, three water gradients were set for plants with mild (65–75% in field water capacity, FC), moderate (55–65% in FC), and severe (45–55% in FC) deficits, respectively. In contrast, an adequate water supply (75–85% in FC) during the growth period was designed as the control (CK). The net photosynthetic rate (Pn), transpiration rate, and stomatal conductance of woad significantly decreased (*P*< 0.05) by moderate and severe water deficits. Still, rehydration after the water deficit could produce a noticeable compensation effect. In contrast, malondialdehyde and proline accumulation significantly increased under moderate and severe water deficits. At the same time, the superoxide dismutase, peroxidase, and catalase all had high activities (increased significantly by 19.87–39.28%, 19.91–34.26%, and 10.63–16.13% compared with CK, respectively), but yields were substantially lower, compared to CK. Additionally, the net photosynthetic rate was negatively correlated with antioxidant enzyme activity. The economic yield of plants subjected to continuous mild water deficit during both vegetative and fleshy root growth was not significantly different from that in CK. Still, the water use efficiency improved significantly. Therefore, the continuous mild water deficit during vegetative and fleshy root growth could improve the physiological and biochemical mechanisms of the plant, representing an optimal irrigation strategy for woad in cold and arid areas.

## 1 Introduction

Water shortage is a serious ecological problem critical to limiting crop growth and production (Rampino et al., 2006; Monreal et al., 2007; Gui et al., 2021; Sheteiwy et al., 2021a; Sheteiwy et al., 2021b; Sheteiwy et al., 2022). Plant water status, photosynthesis, productivity, and water utilization are affected by soil moisture and irrigation systems (Hamoud et al., 2019). Therefore, to overcome the water scarcity problem, efficient agricultural water conservation technologies are urgently needed to enhance water utilization in sustainable crop production (Kang et al., 2002). However, regulated deficit irrigation (RDI) is an irrigation strategy that ensures optimal water status during the phenological period when the crop is most sensitive to water stress and limits other stages (Chai et al., 2016; Galindo et al., 2018). The RDI strategy reduces crop evapotranspiration and can improve irrigation management for null or low yield losses (Carbonell-Barrachina et al., 2015; Lahoz et al., 2016). In addition, mulched drip irrigation is an excellent water-saving technology with the advantages of improving the yield and utilization efficiency of water and fertilizer, weed control, labor input saving, soil erosion reduction, and so on. This technology has been widely used to allow crop cultivation in arid and semiarid areas. It has been shown in previous studies that irrigation combined with plastic film mulching reduced vegetative growth and soil evaporation, improved soil water storage, and increased crop yield and water productivity (Fabeiro et al., 2001; Du et al., 2010). Research on deficit irrigation has been extensively conducted on many foods and cash crops to maintain their productivity and economic benefits in water-scarce areas. Regulated deficit irrigation can save a large amount of irrigation water, reduce crop evapotranspiration, maintain or increase crop yield, and improve crop quality under limited water resources (Patane et al., 2011; Santesteban et al., 2011; Chai et al., 2016; Lahoz et al., 2016).

Photosynthesis is the basis of dry matter formation and the primary determinant of plant yield increases. At the same time, water is an essential raw material required for photosynthesis. Photosynthesis can reflect the response of plants to variable soil moisture levels (Gaju et al., 2016). Changes in photosynthetic rate are closely related to changes in soil microclimates (Fan et al., 2019; Gyimah et al., 2020; Li et al., 2021). Suitable soil water content has a positive effect, while a severe water deficit harms plant photosynthesis (Reich et al., 2018). Plants have evolved mechanisms to rapidly sense stress, actively regulate their stress resistance responses, and address various environmental stresses. As the most prevalent stress factor among abiotic stresses, water stress is vital in limiting crop yield (Bodner et al., 2015). Plants are usually subjected to many physiological and biochemical changes after water stress. Water stress decreases the photosynthetic capacity and causes a significant accumulation of reactive oxygen species (ROS) in crops, causing photooxidative effects and structural damage (Brito et al., 2019). Lipid peroxidation, membrane deterioration, and DNA modification are caused by ROS (Gao et al., 2021; Sheteiwy et al., 2022), negatively affecting crop growth and yield formation (Hao et al., 2019).

Plant tissues contain ROS-scavenging enzymes to control ROS levels and protect cells from stressful conditions (Mittler, 2002; Lv et al., 2022). Additionally, biosynthesis and accumulation of low molecular weight organic compounds, such as osmoregulation, are critical for sustaining osmotic potential under stress (Chaves et al., 2003). Crops improve their adaptability to water stress by accumulating organic solutes, such as proline, soluble carbohydrates, and sucrose (Shao et al., 2005; Zhang et al., 2009; Marcinska et al., 2013; Ahmad et al., 2018).

*Isatis indigotica* (woad) is the basal plant of Daqinye (woad leaves), Banlangen (woad root), and one of the basal plants of Qingdai. Woad is used to cure encephalitis B, mumps, influenza virus, epidemic cerebrospinal meningitis, bacterial and viral infections, and to prevent and control fatal severe acute respiratory syndrome (Tang and Eisenbrand, 1992; Zhu, 1998; Han et al., 2011; Kim et al., 2012; Zhang et al., 2021). Woad is cultivated mainly in northern Chinese regions. It has less strict requirements for light, heat, temperature, and other natural environments. Due to its drought-tolerant characteristics, this species is more suitable for planting in well-drained sandy soils and loam. Minle County of Gansu Province is in the middle of the Hexi Corridor and is located in a typically irrigated agricultural area. Therefore, the cultivation of woad is mainly irrigated. Due to the lack of scientific guidance on irrigation period and amount, traditional flood irrigation technology causes water resource waste, affecting the growth of woad, influencing economic benefits, and resulting in disproportionate input and output.

It is essential to study physiological processes, such as drought resistance and the rehydration recovery mechanism, to enhance the water productivity of woad. Previous studies on woad were mainly focused on its pharmacological effects and chemical composition. Still, there was a low focus on the physiological response of woad to water deficit and rehydration. Moreover, studies on the osmoregulatory response and antioxidant enzyme activities of woad leaves were also scarce. Accordingly, the focus of this study was to investigate the photosynthetic characteristics, the response of the antioxidant protection system, and the osmotic protection function of woad under different water deficit and rehydration conditions. We hypothesized that this study would: (1) investigate the effects of water stress at different reproductive stages on photosynthetic response mechanisms, antioxidant defense mechanisms, and the yield of woad plants and (2) determine the optimal degree and duration of water stress to provide a theoretical foundation for optimal woad cultivation.

## 2 Materials and methods

### 2.1 Site description

**Figure 1:**
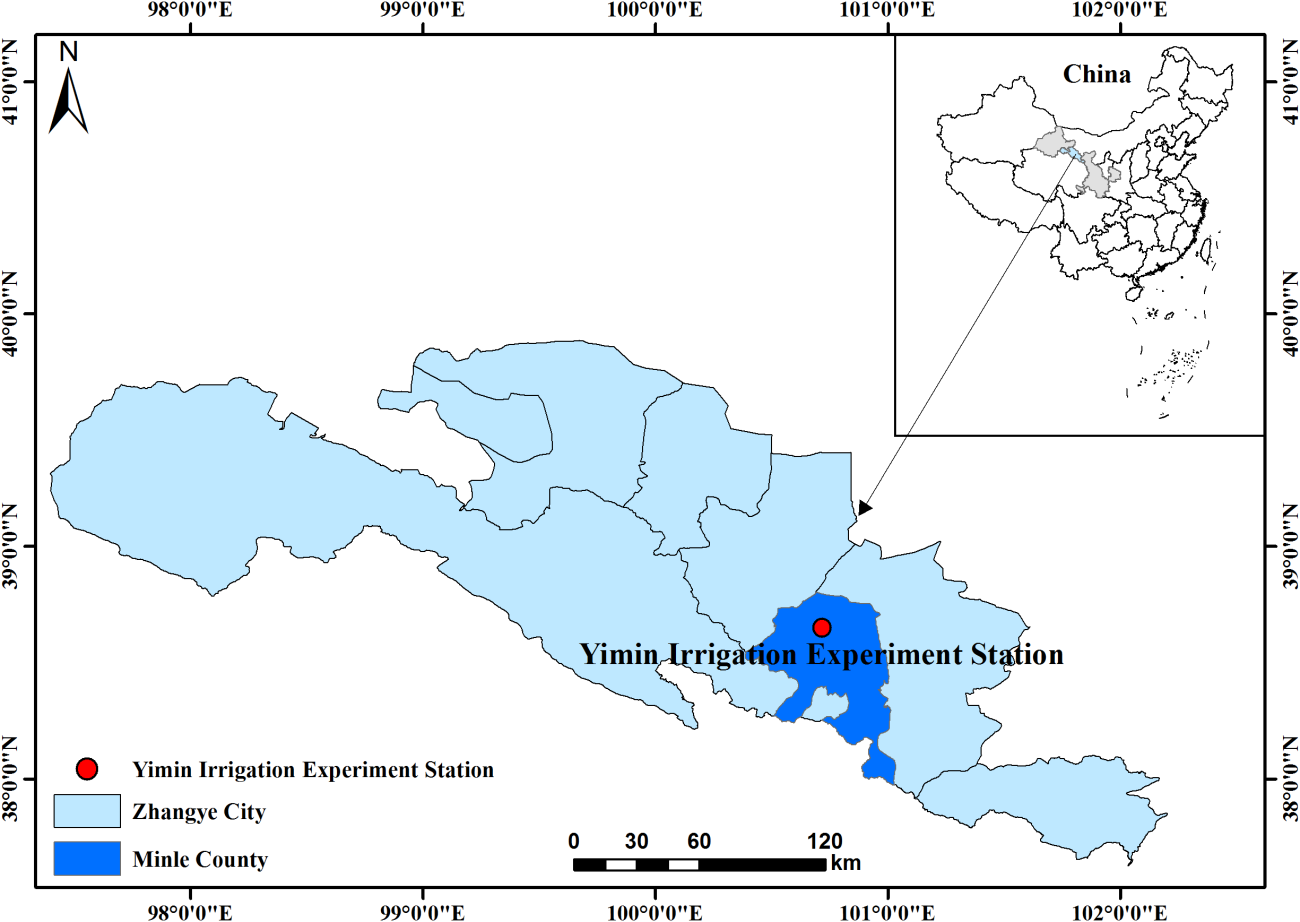
Location of experimental site (Minle, China).

The field experiment was conducted in 2019 at Yimin Irrigation Experiment Station (38°39′N, 100°43′E, mean altitude 1970 m), Minle County, Zhangye City, Gansu Province (**Figure 1**). The experimental station was located in the middle of the Hexi Corridor of Gansu Province, which belonged to the continental desert steppe climate. The annual sunshine time and dryness were 2,932 h and 5.85, respectively. The average annual temperature, evaporation, and rainfall were 7.6 °C, 1,638 mm, and 183–345 mm, respectively. The basic climatic information obtained during the experiment is shown in **Table 1**. Agricultural soil was light loamy soil. The content of organic matter was 12.4 g·kg^-1^. The contents of alkali-hydrolyzed nitrogen, available phosphorus, and available potassium were 57.3 mg·kg^-1^, 15.9 mg·kg^-1^, and 191.7 mg·kg^-1^, respectively. The soil bulk density and field water capacity in the 0–100 cm soil layer was 1.46 g·cm^-3^ and 24 cm^3^·cm^-3^, respectively.

**Table 1:** The basic climatic information in 2019.

Month	T_min_ (°C)	T_max_ (°C)	T_average_ (°C)	RH (%)	Ws (m·s^-1^)	Rainfall (mm)
May	-1.0	26.2	12.6	32.3	1.9	17.1
June	9.1	28.4	17.4	59.61	1.5	75.1
July	8.0	31.6	18.7	57.97	1.5	24.4
August	10.5	32.7	18.8	55.43	1.6	21.9
September	4.7	29.1	14.4	61.2	1.5	63.1
October	-6.4	20.8	6.3	40.3	1.4	8.9

T_min_: minimum air temperature (°C); T_max_: maximum air temperature (°C); T_average_: average air temperature (°C); RH: average relative humidity (%); Ws: wind speed (m·s^-1^).

### 2.2 Experimental materials

Woad was planted in open fields. It was sown on May 4th and harvested on October 10th, 2019. The sowing density was 830,000 plants per hectare, and the size of each plot was 2.7 m × 5 m. The experimental land was mechanically tilled and weeded before sowing. A total of 220 kg·ha^-1^ of urea (46% N content), 330 kg·ha^-1^ of calcium superphosphate (12% P_2_O_5_, 10% S, and 16% Ca content), and 120 kg·ha^-1^ of potassium fertilizer (60% K_2_O content) were applied as base fertilizer. Three drip irrigation belts were installed per plot and covered with colorless mulch.

### 2.3 Experimental design

**Figure 2:**
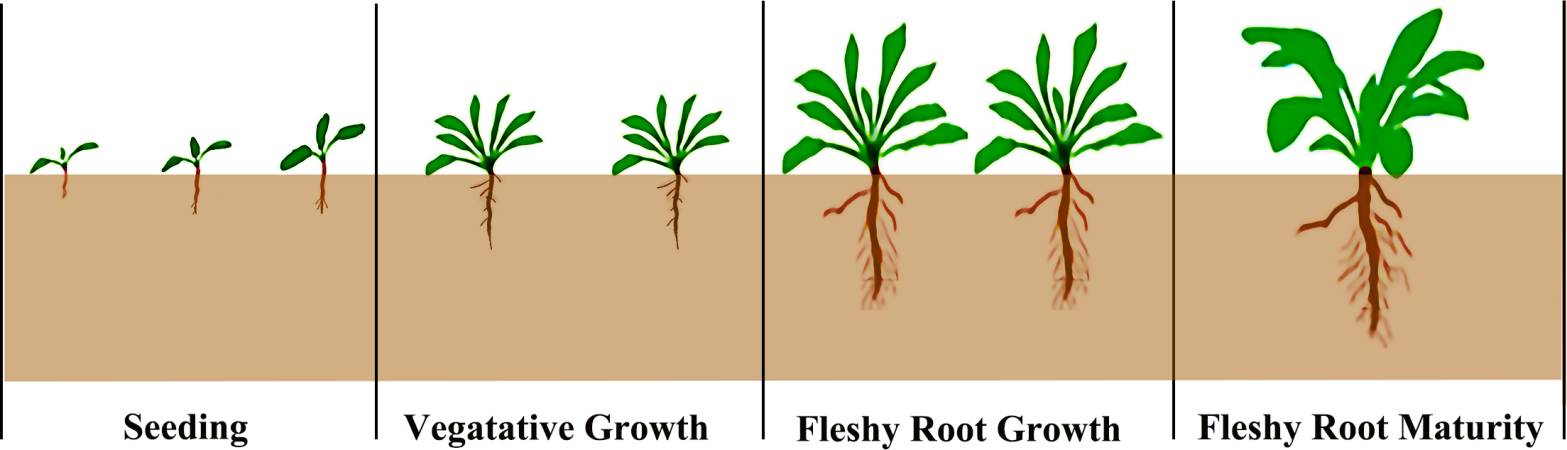
Schematic diagram of the growth period division of woad.

The woad growth stage can be divided into four different growth stages (**Figure 2**): seedling stage (May 4th - June 8th), vegetative growth stage (June 9th - July 20th), fleshy root growth stage (July 21st - August 30th), and fleshy root maturity stage (August 31th - October 10th). For soil moisture, an adequate water supply treatment (75–85% field water capacity, FC) and three water deficit treatments [mild water deficit (65–75% FC), moderate water deficit (55–65% FC), and severe water deficit (45–55% FC)] were set up. Since all growth stages of woad are affected by water deficit, six water stress conditions were established under limited irrigation treatments. Adequate irrigation treatment was used as a control (CK) during the growth period (**Table 2**). The experiment was a single-factor randomized trial with three replicates per treatment and 21 plots. The irrigation amount was measured using a water meter, and soil moisture was contained to the design level. Before sowing, the soil moisture in the 0–100 cm soil layer was adjusted to about 85% of the field water capacity.

**Table 2:** Experimental design.

Treatments	Seeding	Vegetative growth	Fleshy root growth	Flesh root maturity
T1	75–85%	65–75%	75–85%	75–85%
T2	75–85%	55–65%	75–85%	75–85%
T3	75–85%	45–55%	75–85%	75–85%
T4	75–85%	65–75%	65–75%	75–85%
T5	75–85%	55–65%	55–65%	75–85%
T6	75–85%	65–75%	75–85%	65–75%
CK	75–85%	75–85%	75–85%	75–85%

### 2.4 Measurements and calculations

#### 2.4.1 Photosynthetic physiological and ecological indicators

The changes in the net photosynthetic rate (Pn), stomatal conductance (Gs), and transpiration rate (Tr) of woad were measured using a portable photosynthetic measurement system (LI-6400, LI-COR, USA) from 9:30 am to 10:30 am in sunny and cloudless weather (measured on June 7, July 19, August 28 and October 7, respectively). The water use efficiency of woad plants (WUEL) was calculated as the ratio of the Pn to the Tr.

#### 2.4.2 Malondialdehyde

MDA was determined using the method proposed by Li et al. (2010). Fresh woad leaves were weighed (0.5 g) and ground with 2 ml of 10% trichloroacetic acid (TCA) and a little quartz sand until the sample was homogenized. The homogenate was ground by adding 8 mL of 10% TCA and centrifuged at 4000 r·min^-1^ for 10 min. A total of 2 ml was taken from the supernatant. The reference was 2 ml of distilled water. A total of 2 ml of 0.6% thiobarbituric acid (TBA) was added, mixed well, boiled in a water bath at 100 °C for 15 min, cooled rapidly, and then centrifuged. The absorbance values of the supernatants at 450 nm, 532 nm, and 600 nm were measured. The MDA content was calculated using the following formula:


$$MDA(\mu mol\cdot g^{-1})=\frac{\left[6.45\times (D532-D600)-0.56D450\right]\times Vt}{V_{1}\times Fw}$$

where Vt was the total volume of the extract (ml), V_1_ was the volume of the reactant (ml), and Fw was the mass of the fresh sample (g).

#### 2.4.3 Proline

Pro was determined using the acidic ninhydrin method (Bates et al., 1973). Fresh woad leaves were weighed (0.3 g). A total of 5 ml of sulfosalicylic acid was added, boiled in a water bath for 10 min, and filtered. A total of 2 ml of filtrate was aspirated with 2 ml of glacial acetic acid and acidic ninhydrin mixture. The sample was boiled in a water bath for 40 min and cooled. Then, 4 ml of toluene was added, and the sample was thoroughly shaken. After static stratification, the supernatant was taken for colorimetry at 520 nm.

#### 2.4.4 Leaf antioxidant enzyme activity

##### 2.4.4.1 Enzyme solution preparation

Fresh woad leaves were weighed (0.5 g) into a mortar, poured into a centrifuge tube with 5 ml phosphate buffer (pH 7.8), and ground in an ice bath. The sample was cryogenically centrifuged for 20 min at 10,000 rpm. The supernatant (enzyme solution) was poured into test tubes and stored at 0–4°C until use, and used for the enzyme assay.

##### 2.4.4.2 Measurement of superoxide dismutase (SOD), peroxidase (POD), and catalase (CAT) activities

SOD was determined using the method proposed by Tewari et al. (2006). POD was determined using the method proposed by Nakano and Asada (1981). CAT was determined using the method proposed by Dhindsa et al. (1981).

#### 2.4.5 Yield and water use efficiency

When woad plants were mature, 1 m × 1 m sample squares were selected to extract woad and then weighed to calculate the yield. WUE was calculated using the following formula:


$$WUE=\frac{Y}{ET}$$

where WUE was the water use efficiency of woad (kg·ha^-1^·mm^-1^), Y was the yield of woad (kg·ha^-1^), and ET was the total water consumption (mm) of woad during the entire growth period.

### 2.5 Data analysis

Data were processed using Microsoft Excel 2013 and plotted using Origin 2021. The data were analyzed by one-way analysis of variance (ANOVA) with IBM SPSS 23.0, and the least significant difference (LSD) was compared between the mean values of each treatment when the P value ≤ 0.05. A linear function was used to fit the model between Pn and antioxidant enzyme activity at different growth stages.

## 3 Results

### 3.1 Effect of water deficit on the photosynthesis of woad

The Pn, Tr, and Gs had a single-peaked curve variation, with an increasing and then decreasing trend in the entire woad growth stages (**Table 3**). The Pn and Gs in each water deficit treatment peaked at the fleshy root growth stage, while Tr peaked at the vegetative growth stage. The photosynthetic parameters (Pn, Tr, and Gs) increased the most in the vegetative growth stage and decreased the most in the fleshy root maturity stage. The changes in the Pn, Tr, and Gs of leaves were not significantly (*P* > 0.05) different among treatments applied to seedlings. In vegetative growth, Tr and Gs of plants in the T4 treatment were the highest and increased significantly (*P*< 0.05) by 16.96% and 26.04%, respectively, compared with CK. During vegetative growth, the leaf Pn of individuals in CK was the largest. No significant differences were seen in leaf Pn among individuals in the T1, T4, and T6 treatments during the vegetative growth, which was significantly lower than that of CK. At vegetative growth, the Pn, Tr, and Gs of individuals in the T2 treatment were significantly reduced (*P*< 0.05) by 13.87%, 18.80%, and 15.94%, respectively, compared to CK. At the same time, the Pn, Tr, and Gs of plants in treatment T3 were significantly reduced by 22.08%, 46.28%, and 38.40%, respectively. These indicate that the decrease of Pn, Tr, and Gs increases with the aggravation of water deficit.

**Table 3:** Effects of different water deficits on the Pn, Tr, and Gs of woad leaves.

Indices	Treatments	Seeding	Vegetative growth	Fleshy root growth	Fleshy root maturity
Pn (μmol CO_2_ m^-2^ s^-1^)	T1	7.67a	20.57b	23.79a	15.50a
	T2	7.50a	18.34c	20.93c	12.93d
	T3	7.58a	16.59d	18.40e	11.12e
	T4	7.94a	20.64b	23.57a	15.00b
	T5	7.54a	18.61c	20.17d	12.64d
	T6	7.64a	20.34b	23.44a	13.59c
	CK	7.79a	21.29a	22.17b	15.24ab
Tr (μmol H_2_O m^-2^ s^-1^)	T1	3.72a	16.20ab	14.77a	9.30a
	T2	3.75a	12.10c	10.72bc	7.08c
	T3	3.69a	8.01d	9.79c	6.36d
	T4	3.87a	17.43a	15.24a	8.94a
	T5	3.69a	14.58b	11.04b	6.43d
	T6	3.78a	16.19ab	15.39a	7.74b
	CK	3.86a	14.90b	15.17a	9.04a
Gs (mol H_2_O m^-2^ s^-1^)	T1	0.16a	1.04b	1.33a	0.93a
	T2	0.16a	0.89c	0.92c	0.71c
	T3	0.15a	0.65d	0.84c	0.46d
	T4	0.18a	1.34a	1.35a	0.88a
	T5	0.17a	0.84c	0.84c	0.67c
	T6	0.18a	1.22a	1.27a	0.80b
	CK	0.18a	1.06b	1.11b	0.90a
WUEL	T1	2.06a	1.27cd	1.61b	1.67d
	T2	2.00a	1.52b	1.95a	1.83b
	T3	2.05a	2.07a	1.88a	1.75c
	T4	2.05a	1.18d	1.55b	1.68d
	T5	2.04a	1.28cd	1.83a	1.97a
	T6	2.02a	1.26cd	1.52bc	1.76c
	CK	2.02a	1.43bc	1.46b	1.69d

Different lowercase letters in the same column mean the significant differences among treatments according to LSD (P < 0.05). Data were presented as mean ± SE (n = 3).

The Pn of plants in T1 and T6 during fleshy root growth was significantly enhanced by 7.31% and 5.75%, respectively, and Gs also increased significantly (*P*< 0.05) by 19.23% and 10.15%, respectively, compared to CK. The Pn and Gs of plants in the T2 treatment increased by 3.78% and 9.74% compared with the T5 treatment during fleshy root growth. The Pn, Tr, and Gs of plants in the T3 treatment during fleshy root growth increased compared to those in vegetative growth. This result was an indication that rehydration during fleshy root growth stage produced a compensation effect on plant photosynthesis. After entering the fleshy root maturity stage, woad plants began to senescence. Pn, Tr, and Gs of leaves in each water deficit treatment were obviously reduced compared to fleshy root growth. Pn, Tr, and Gs of T3 at fleshy root maturity were the smallest and significantly lower than CK. Compared to T4, the Pn, Tr, and Gs of plants in the T6 treatment significantly decreased at fleshy root maturity, indicating that the photosynthesis of woad leaves was also affected by the water deficit period.

Water use efficiency of leaves (WUEL) had a parabolic variation of decreasing followed by increasing throughout the reproductive period, except for T3 (**Table 3**). The highest WUEL was found at the seedling stage and no significant difference in WUEL was observed among treatments. The highest WUEL was recorded in the vegetative growth period of T3, with a significant (*P*< 0.05) increase of 44.97% compared to the control CK. The WUEL of plants in the T1, T4, and T6 treatments was lower than CK at vegetative growth, but no significant differences were observed among treatments. The WUEL in T2 during vegetative growth increased (*P* > 0.05) by 6.57% compared to CK. The lowest WUEL was found in plants in CK at fleshy root growth, and WUEL increased in all water deficit treatments compared to CK. Plants in T2, T3, and T5 were significantly different from CK. The remaining treatments were not significantly different from CK. In fleshy root maturity, the WUEL was at the same level in plants in the T1, T4, and CK treatments. T2, T3, T5, and T6 treatments at fleshy root maturity increased significantly by 3.74–16.61% compared to CK. These results indicated that different water deficit levels and periods had different effects on WUEL.

### 3.2 Effects of water deficit on leaf physiological and biochemical indices

#### 3.2.1 Leaf malondialdehyde

**Figure 3:**
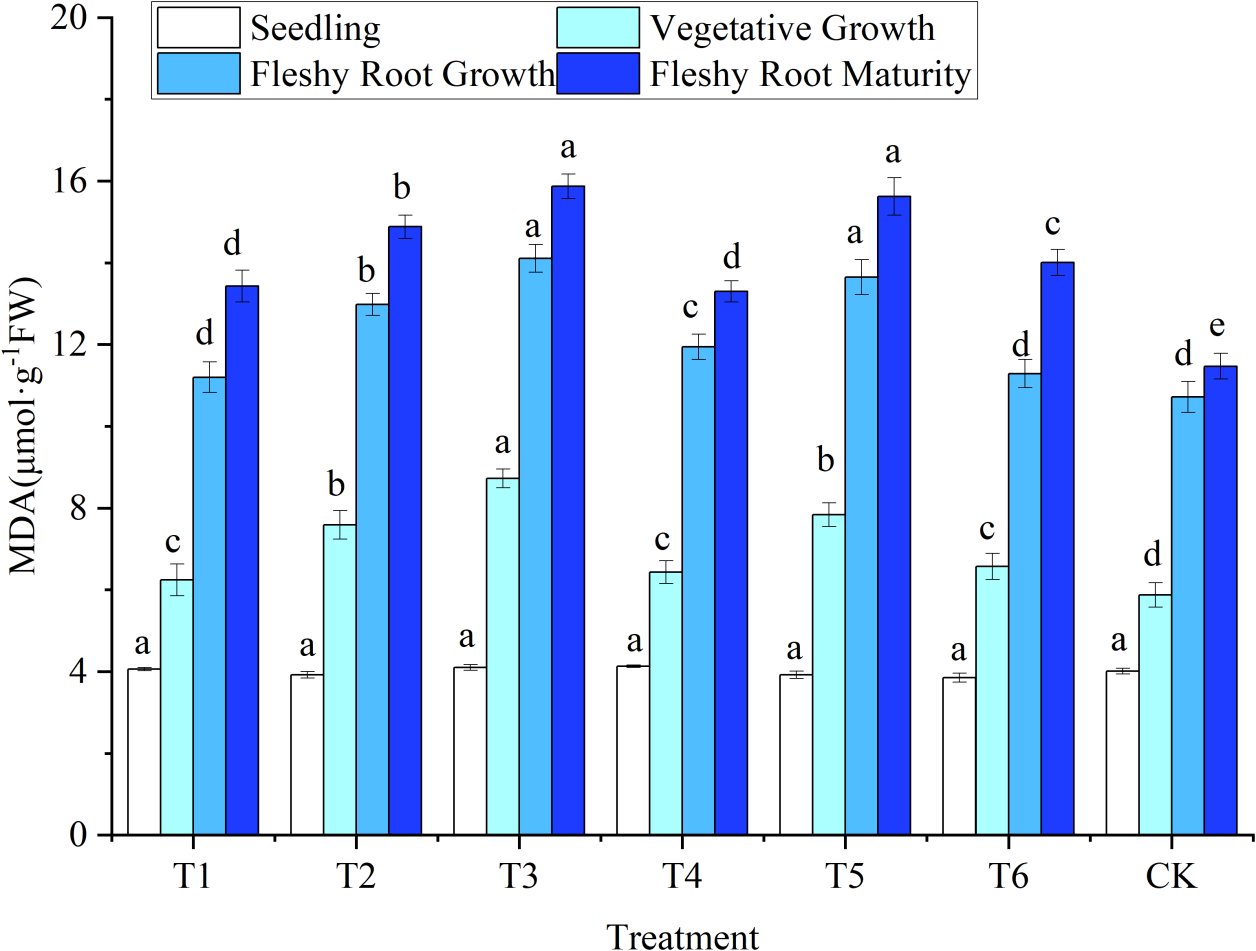
Response of malondialdehyde in woad plants in different water deficits. Different lowercase letters in the same column mean the significant differences among treatments according to LSD (*P*< 0.05). Data were presented as mean ± SE (n = 3).

The MDA content was at the same level (*P* > 0.05) in all treatments at the seedling stage (**Figure 3**). After entering the vegetative growth, the MDA content of CK was the lowest. The MDA content of other treatments increased significantly (*P*< 0.05) by 6.30–48.55%. The MDA content in the T3 treatment was significantly increased by 14.89% compared to the T2 treatment. This result showed that the MDA of woad leaves was increased by the water deficit treatment, and the increase was greater with more extensive water deficits. No significant difference was observed among treatments T1, T6, and CK during fleshy root growth. At fleshy root growth, The MDA content of plants in T1 were reduced by 6.20% compared to T4, while T2 was reduced by 4.91% compared to T5, indicating that rehydration treatment was beneficial in reducing the MDA content in woad leaves. The MDA content of plants in the T1 and T4 treatments decreased significantly by 4.14% and 5.07%, respectively, compared to the T6 treatment. The results showed that the MDA content of woad leaves was affected by both the degree and the period of water deficit.

#### 3.2.2 Leaf proline

**Figure 4:**
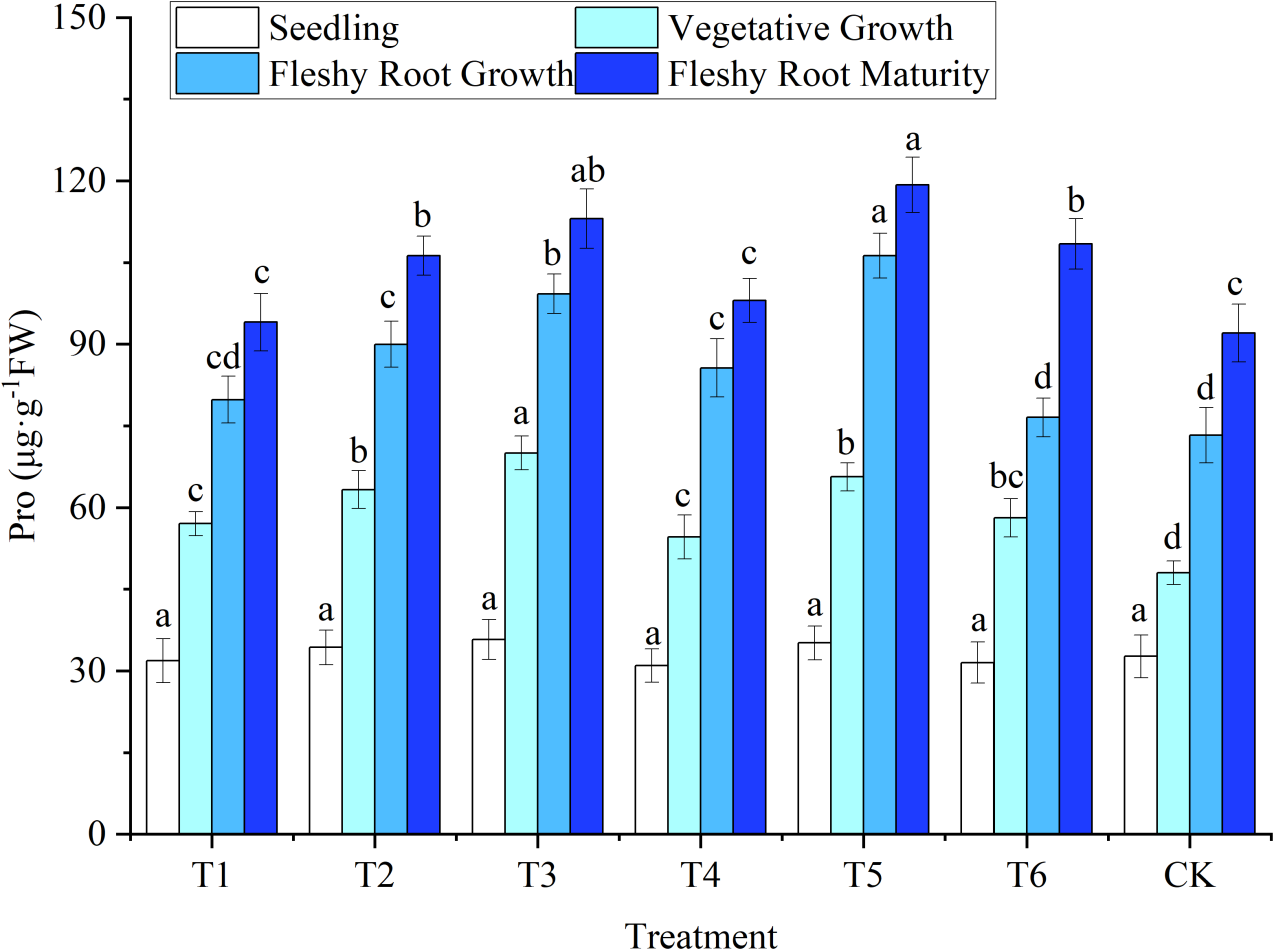
Response of proline in woad plants in different water deficits. Different lowercase letters in the same column mean the significant differences among treatments according to LSD (*P*< 0.05). Data were presented as mean ± SE (n = 3).

Pro content was correlated with the level of plant water stress. The more water is lost by plants, the more Pro they produce. As shown in **Figure 4**, the change patterns of Pro and MDA contents of woad leaves were similar throughout the entire growth period. No significant difference (*P* > 0.05) was found in Pro content among treatments at seedlings. The Pro content of each water deficit in vegetative growth increased significantly (*P*< 0.05) by 13.70–45.81% compared with the CK. The highest Pro content was observed in T3, illustrating that the water deficit increased Pro content in woad leaves. Such an increase was more significant with an increase in the level of water deficit. At fleshy root growth, the Pro content in T1 and T6 treatments decreased compared to T4, while T2 had a significant decrease of 15.31% compared to T5. This indicates that the compensation effect produced by the rehydration treatment was beneficial in reducing the Pro content. At fleshy root maturity, the Pro content of plants in the T1 and T4 treatments returned to levels close to the CK. Meanwhile, Pro content in treatments T3 and T5 was significantly higher than CK, by 22.85% and 29.55%, respectively. This indicates that the rehydration effect after mild water deficit adjustment would reduce Pro content due to its rehydration compensation effect. In contrast, severe water deficit adjustment and long-term moderate water deficit caused severe plant damage, and the rehydration compensation effect was not apparent after rehydration.

#### 3.2.3 Leaf antioxidant enzyme activity

**Figure 5:**
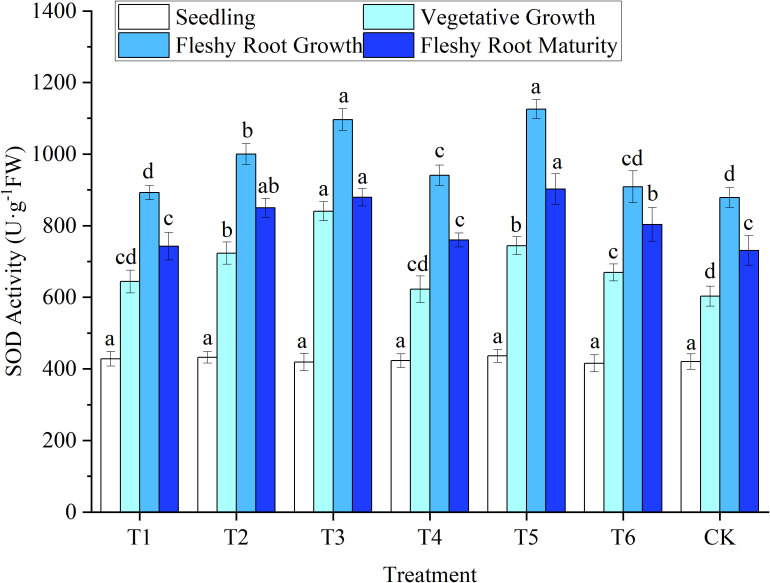
Response of superoxide dismutase activity in woad plants in different water deficits. Different lowercase letters in the same column mean the significant differences among treatments according to LSD (*P*< 0.05). Data were presented as mean ± SE (n = 3).

**Figure 6:**
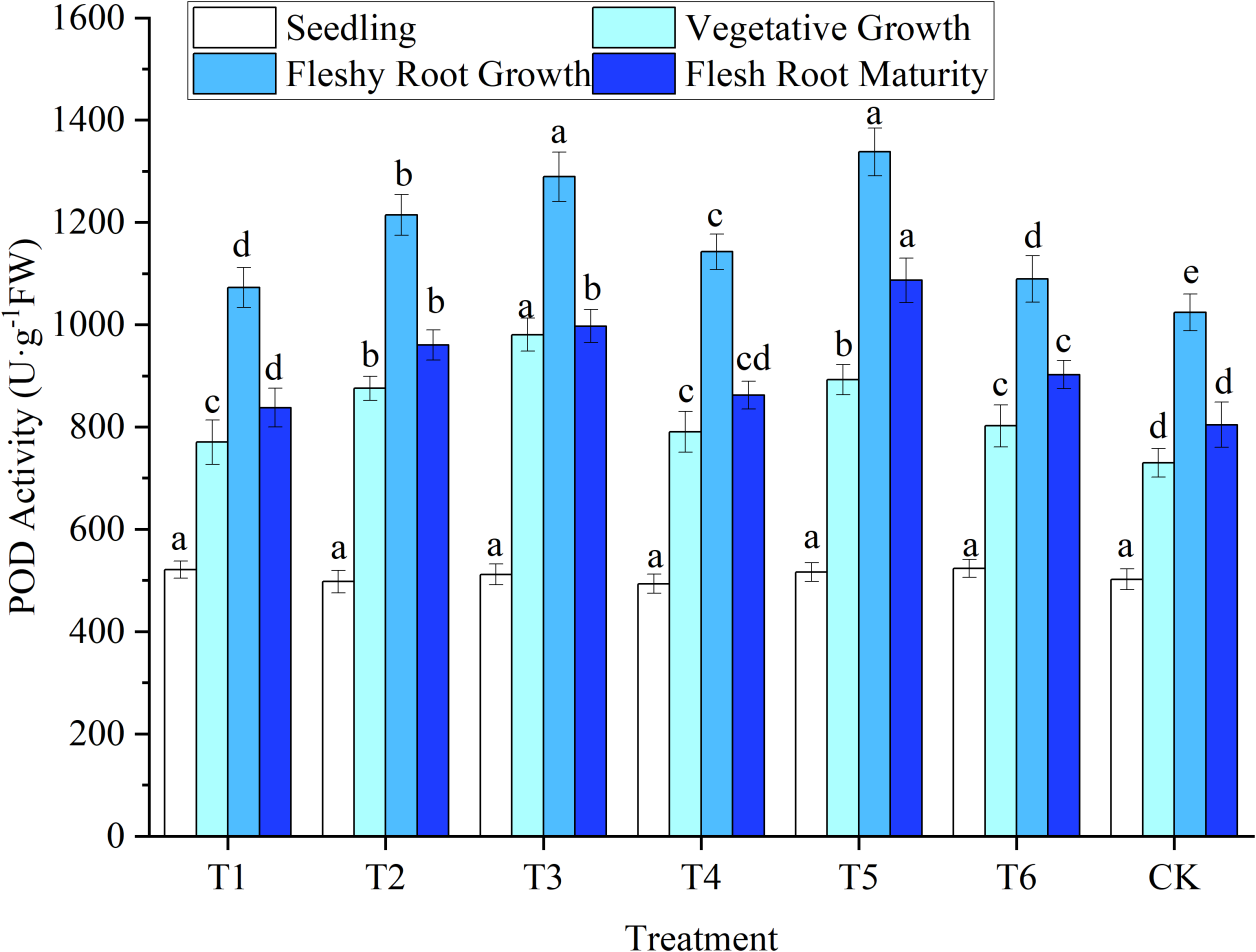
The response of peroxidase (POD) activity in woad plants in different water deficits. Different lowercase letters in the same column mean the significant differences among treatments according to LSD (*P*< 0.05). Data were presented as mean ± SE (n = 3).

**Figure 7:**
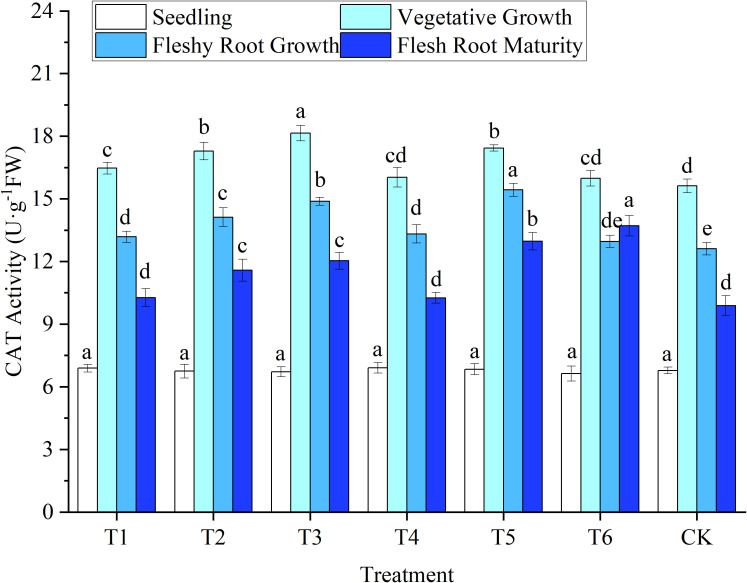
Response of catalase activity in woad plants in different water deficits. Different lowercase letters in the same column mean the significant differences among treatments according to LSD (*P*< 0.05). Data were presented as mean ± SE (n = 3).

The SOD, POD, and CAT activities in woad leaves had an increasing trend at first and then a decreasing trend with the advancement of the growth period. SOD and POD activity increased rapidly to a maximum at fleshy root growth. In contrast, CAT peaked during vegetative growth. At the late growth stage, water deficits or sufficient water supply affected enzyme activity and reduced its ability to removed peroxide (**Figures 5**–**7**).

##### 3.2.3.1 SOD

The SOD activity of woad leaves was lowest in seedlings (**Figure 5**). Still, no significant differences were observed among treatments (*P* > 0.05). The SOD activity of CK leaves was lowest in vegetative growth, while the SOD activity of the other treatments increased. At vegetative growth, the leaf SOD activities of T1 and T4 were increased (*P* > 0.05) compared to CK. In contrast, in the T2 and T3 treatments, SOD activity was significantly enhanced (*P*< 0.05) by 19.86% and 39.28% compared to CK. The SOD activity of T3 at vegetative growth was significantly enhanced by 16.20% compared to T2. This illustrates that with the increase in water deficit, woad plants would have higher SOD activity under their protective mechanism to resist the damage caused by adversity. At fleshy root growth, the SOD activity of the leaves increased to the peak compared with vegetative growth. The SOD activity of T1 was significantly reduced by 5.16% compared to T4. The SOD activity of T2 was significantly reduced (*P*< 0.05) by 11.13% compared to T5, which showed a specific rehydration compensation effect. The SOD activity of woad leaves in all treatments at fleshy root maturity decreased compared to fleshy root growth. This was probably due to the gradual senescence of the plant, which produced excess reactive oxygen species and metabolic dysregulation in the plant. SOD activity did not differ significantly between T1, T4, and CK. Still, the SOD activity of plants in the T6 treatment increased significantly by 8.21% compared to plants in the T1 treatment.

##### 3.2.3.2 POD

The changes in the POD and SOD activities of woad plants were similar (**Figure 6**). No significant differences (*P* > 0.05) were observed in POD activity among all treatments at seedlings. In vegetative growth, the POD activity of the leaves of plants in the T3 treatment was the highest, with a significant increase of 34.26% compared to CK. In vegetative growth, the POD activity of the T2 and T5 leaves increased significantly (*P*< 0.05) by 19.91% and 22.23% compared to CK, respectively. In contrast, the POD activity in T3 was significantly higher (*P*< 0.05) than that of T2 and T5. This indicates that with the increase in the water deficit, the POD enzyme activity in woad plants will be enhanced with the increase of SOD activity. Jointly, this may eliminate the excess ROS in the plant to resist the damage caused by drought. At fleshy root growth, the POD activity values of each treatment reached the maximum value, and the POD activity of T1 decreased (*P*< 0.05) by 6.14% compared to T4. At the same time, the POD activity of T2 was reduced (*P*< 0.05) by 10.80% compared to T5. Rehydration after water deficit could significantly reduce POD activity in leaves. The POD activity in the leaves of plants in all treatments in fleshy root maturity was significantly lower than that in fleshy root growth. Still, no significant differences were observed in POD activity among the plants in the T1, T4, and CK treatments. This illustrates that the POD activity recovered to a similar level of CK after rehydration in mild water deficit.

##### 3.2.3.3 CAT

The lowest CAT activity was observed in the leaves of woad seedlings, but no significant differences (*P* > 0.05) among treatments (**Figure 7**). After entering the vegetative growth stage, leaf CAT activity increased rapidly, reaching a peak. Still, the lowest CAT activity was found in CK, while CAT increased in other water deficits compared to CK. During vegetative growth, the CAT activity of T3 significantly improved by 16.13% compared to CK. At the same time, the CAT activity of the T2 and T5 treatments increased significantly by 10.63% and 11.59%, respectively, compared to CK. Compared with the vegetative growth stage, the activity of CAT decreased significantly (*P*< 0.05) in fleshy root growth. No significant difference in CAT activity between T6 and CK was observed at fleshy root growth. In contrast, the CAT activity of the T2 treatment was significantly reduced by 8.49% compared to T5, indicating that the rehydration treatment would produce a specific compensation effect. No significant difference in CAT activity at fleshy root maturity among the T1, T4, and CK treatments. Still, the leaf CAT activity in T6 increased significantly by 3.89% compared with CK. The CAT activity of the woad leaves was increased by a mild deficit treatment at the fleshy root maturity stage.

### 3.3 Effects of water deficit on yield and WUE in woad

**Figure 8:**
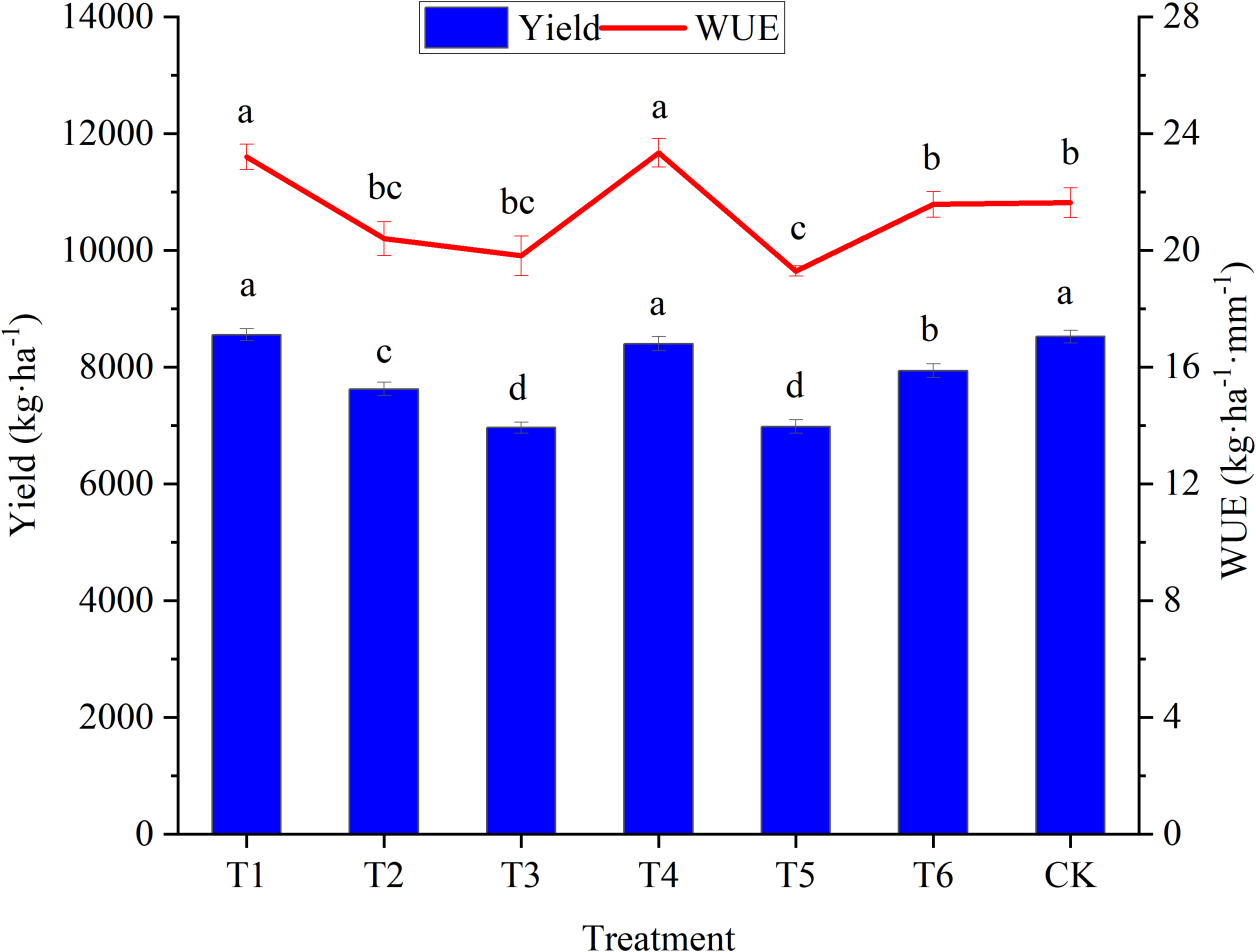
Response of yield and water use efficiency in different water deficit treatments. Different lowercase letters mean the significant differences among treatments according to LSD (*P*< 0.05). Data were presented as mean ± SE (n = 3).

The yield of woad in the T1 and T4 treatments did not decrease compared to CK (**Figure 8**). WUE in treatments in plants in the T1 and T4 was enhanced significantly (*P*< 0.05) by 7.84% and 6.92%, respectively, compared to CK. This demonstrated that a mild water deficit at the vegetative and fleshy root growth periods improved the WUE without significantly affecting the woad yield. The WUE of plants in the T6 treatment was not reduced significantly (*P* > 0.05) compared to CK. Nevertheless, the yield was significantly reduced by 6.74%. The economic yield of woad in the other water deficits decreased significantly by 9.80–17.74% compared to CK. WUE of plants in the T5 treatment was reduced significantly (*P*< 0.05) by 10.24% compared to CK. It was shown that significant decreases in the yield was caused by moderate and severe water deficits, and significant decreases in the WUE was caused by severe water deficits, with decreases being more extensive with the water deficit degrees. Both yield and WUE were significantly lower in T6 compared to plants in the T1 and T4 treatments. This meant that the water deficit period also influenced the yield and WUE of woad.

### 3.4 Correlation analysis

#### 3.4.1 Correlations between Pn and antioxidant enzyme activity

**Figure 9:**
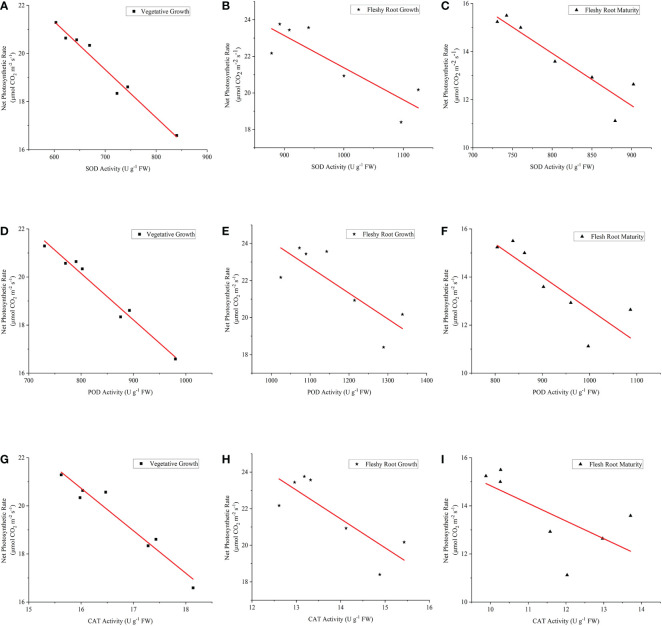
The relationship between Pn and the antioxidant enzyme activities of woad. **(A–C)** describe the relationship between Pn and SOD in vegetative growth, fleshy root growth and fleshy root maturity, respectively. **(D–F)** describe the relationship between Pn and SOD in vegetative growth, fleshy root growth and fleshy root maturity, respectively. **(G–I)** describe the relationship between Pn and SOD in vegetative growth, fleshy root growth and fleshy root maturity, respectively. Pn: the net photosynthetic rate; SOD, superoxide dismutase; POD, peroxidase; CAT, catalase.

It is shown in **Figure 9** (A–I) that there were significant and negative linear correlations between An and other antioxidant enzyme activity (*P*< 0.05). As for the fitting equation of SOD activity and Pn (**Table 4**), the slopes of the three growth stages were consistent with the advancement of the growth process. The intercept tended to increase and then decrease, while R^2^ (coefficient of determination) tended to decline and then increase. It was also shown that the POD activity and Pn fitting equations had a similar trend. However, the slope, intercept, and R^2^ of the fitting equation for CAT activity and Pn decreased with the advancement of the growth stage. The highest dispersion was observed in the fitting equations for SOD activity against Pn and POD activity against Pn at fleshy root growth. At the same time, the highest dispersion was observed in the fitting equations for CAT activity and Pn during the fleshy root maturity period.

**Table 4:** The fitting equation, coefficient of determination (R^2^), and residual sum of squares (RSS) of Pn and antioxidant enzyme activity of woad under water deficit at different growth stages.

Indexes	Growth stage	Fitting equation	R^2^	RSS
Pn and SOD	Vegetative growth	Y_V_ = -0.02X_VSOD_ + 33.31	0.968^**^	0.539
	Fleshy root growth	Y_F_ = -0.02X_FSOD_ + 38.90	0.741^*^	6.407
	Flesh root maturity	Y_M_ = -0.02X_MSOD_ + 31.33	0.852^**^	2.316
Pn and POD	Vegetative growth	Y_V_ = -0.02X_VPOD_ + 35.62	0.976^**^	0.407
	Fleshy root growth	Y_F_ = -0.01X_FPOD_ + 38.00	0.642^*^	8.880
	Flesh root maturity	Y_M_ = -0.01X_MPOD_ + 26.29	0.701^*^	4.682
Pn and CAT	Vegetative growth	Y_V_ = -1.77X_VCAT_ + 49.00	0.944^**^	0.955
	Fleshy root growth	Y_F_ = -1.58X_FCAT_ + 43.49	0.667^*^	8.243
	Flesh root maturity	Y_M_ = -0.73X_MCAT_ + 22.19	0.448^*^	8.663

Y_V_, Y_F,_ and Y_M_ represent the net photosynthetic rate during the vegetative growth period, fleshy root growth period, and fleshy root maturity period, respectively. X_VSOD_, X_FSOD_, and X_MSOD_ represent SOD activity during the vegetative growth, fleshy root growth, and fleshy root maturity periods. X_VPOD_, X_FPOD_, and X_MPOD_ represent the POD activity during the vegetative growth period, fleshy root growth period, and fleshy root maturity period, respectively. X_VCAT_, X_FCAT_, and X_MCAT_ represent CAT activity during the vegetative growth, fleshy root growth, and fleshy root maturity periods. * and ** mean that the R^2^ is significant at the probability levels of 0.05 and 0.01, respectively.

#### 3.4.2 Correlations among leaf photosynthetic parameters, physiological and biochemical indices, yield, and WUE

**Figure 10:**
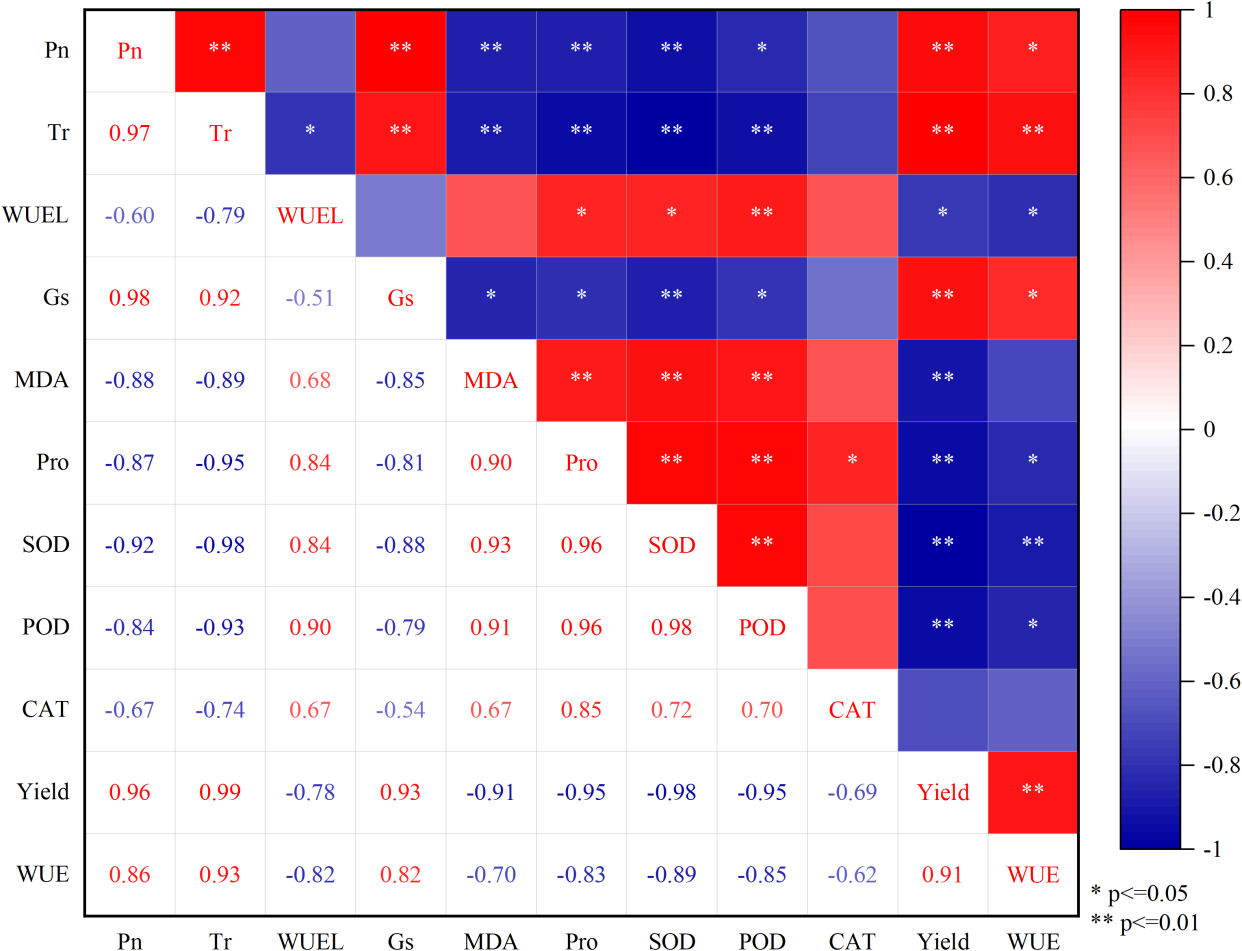
The correlations among Pn, Tr, WUEL, Gs, MDA, Pro, SOD, POD, CAT, yield, and WUE. The numbers in the squares are the correlation coefficients. Pn, the net photosynthetic rate; Tr, transpiration rate; WUEL, the water use efficiency of woad leaves; Gs, stomatal conductance; MDA, malondialdehyde; Pro, proline; SOD, superoxide dismutase; POD, peroxidase; CAT, catalase; WUE: water use efficiency.

The correlations between Pn, Tr, WUEL, Gs, MDA, Pro, SOD, POD, CAT activity, yield, and WUE are analyzed in detail in **Figure 10**. The Pn, Tr, Gs, and WUE were positively correlated with yield (*P*< 0.01). In contrast, MDA, Pro, SOD activity, and POD activity were negatively correlated with yield (*P*< 0.01). Also, MDA, Pro, and SOD were negatively correlated with Pn (*P*< 0.01), and POD activity was negatively correlated with Pn (*P*< 0.05). Pro, SOD activity, and POD activity were positively correlated with MDA (*P*< 0.01). In addition, the SOD activity was positively correlated with POD activity (*P*< 0.01), indicating a similar response trend of these two indicators to water deficit.

## 4 Discussion

Photosynthesis is the most fundamental biological phenomenon in plants and has the best sensitivity to water stress (Bano et al., 2021). Leaves are vital photosynthetic organs, and the photosynthetic capacity of leaves is directly affected by changes in water content (Acreche et al., 2009). The accumulation of dry matter and yield was closely related to the variation in the photosynthesis parameters of leaves. At the same time, water deficits and rehydration interfere with the natural processes of photosynthesis in single leaves. It is well known that water deficits cause diminished photosynthesis, affecting crop productivity and yield (Guerfel et al., 2009). It was revealed that the photosynthesis of woad was affected by different water deficits at different periods. This study found that the Pn, Tr, and Gs of woad decreased significantly with moderate and severe water deficits. This was similar to the findings of Ma et al. (2015), where deficit irrigation reduced Pn, Gs, and Tr, while the reduction in Pn was mainly caused by the decrease in Gs. Consequently, there was a reduction in the intercellular carbon dioxide concentration. In contrast, there was no significant decrease in Pn, Tr, and Gs during vegetative growth under a mild water deficit compared to CK. This result indicates that an appropriate mild water deficit would not obviously inhibit photosynthesis. This was similar to the results found by Fan et al. (2019). They showed that proper soil moisture could enhance crop canopy structure, which was the foundation for increased photosynthetic and dry matter accumulation.

This study indicated that the decrease in Pn caused by the water deficit could be recovered by the compensatory effect of rehydration treatment. The Pn of moderate and severe water deficit continued to decrease with increasing water deficit before rehydration treatment. In contrast, Pn increased with the rehydration treatment (Li et al., 2019). The decrease in Pn after the water deficit may be due to the reduction of stomata caused by the decline in carbon dioxide diffusion and internal carbon dioxide concentration. After rehydration, Gs and internal carbon dioxide concentrations returned to similar levels as before rehydration. Compared with T1, the Pn, Tr, and Gs of the leaves of plants in the T6 treatment at the fleshy root maturity period were significantly lowered by 12.35%, 16.70%, and 13.82%, respectively. This result indicates that woad photosynthesis was affected by the level and period of water deficit. The irrigation quantity and irrigation period affected the photosynthesis of the crop (Liu et al., 2016). In this study, moderate and severe water deficits were beneficial in improving woad WUEL. Pn and Tr were reduced by water deficit compared to adequate irrigation, increasing the WUEL by 2.7–26.1% (Cui et al., 2009). The WUEL under deficit irrigation treatment was higher than under conventional irrigation treatment (Li et al., 2018). Water deficit led to a simultaneous reduction in Pn and Tr, but increased WUEL because of a more significant decrease in Tr. Shao et al. (2011) similarly concluded that water deficit treatment slightly decreased Pn and Tr, increasing WUEL by 24–26%.

When plants suffer water stress, ROS accumulate, causing peroxidation reactions of cell membrane lipids. MDA is one of the peroxidation products of membrane lipids. MDA content can determine peroxidation levels, reflecting the level of plant damage under stress (Pravisya et al., 2019). It was shown in this study that the MDA content of plants in water deficit increased significantly during vegetative growth (*P*< 0.05), from 6.30% to 48.55% compared to CK. Such an increase was more extensive with the degree of water deficit. MDA content increased in all drought stress treatments (Cao et al., 2022). The full irrigation treatment of water-stressed Aloe vera plants with low MDA content reduced protein levels but improved MDA values (Khajeeyan et al., 2019). The MDA content of wheat increased significantly after 10 days of drought stress (Abid et al., 2018). Still, the MDA content and antioxidant enzyme activities could be restored to control levels after rehydration in moderate drought. Nonetheless, the values under severe drought were still higher than in CK (Abid et al., 2018). Generally, these results were consistent with our results, in which the MDA content of woad leaves was significantly reduced after rehydration during fleshy root growth.

When plants suffer water stress, cell water loss was severe. A variety of self-reaction mechanisms are adopted to improve adaptability to stress and maintain the osmotic balance of the cell, including the accumulation of osmotic regulators and enhancement of antioxidant enzyme activity. Pro regulates intracellular osmotic potential and stabilizes the cell membrane structure in water stress (Huseynova et al., 2016). We found that Pro content in woad leaves may be increased by water deficit. This result may be due to the damage to stressed cells caused by the water deficit. Therefore, stressed cells are protected by Pro, and the osmotic potential between cells is regulated by Pro (Heerden and Kruger, 2002). This was consistent with research involving winter wheat. It was found that water stress enhanced the Pro concentration in winter wheat and that Pro was mainly involved in the protective effect of oxidative stress rather than osmoregulation in the early stages of water stress (Tatar and Gevrek, 2008). It was found in this study that the Pro content of plants in severe water deficits was the highest in the vegetative growth stage. During this growth stage, plants suffered severe oxidative damage, and their leaves became yellow and fell off easily from the plant. This was also consistent with the results found for *Scutellaria baicariae*. Mild and moderate drought stress treatments increased MDA, Pro, and soluble protein contents. Also, the Pro content peaked under severe drought stress (Cheng et al., 2018).

SOD, POD, and CAT are essential constituents of the non-enzymatic antioxidant system in plants (Helaly et al., 2017). SOD rapidly converts ${\text{O}}_{2}^{-}$ and H_2_O_2_ through a displacement reaction (Quan et al., 2008). POD removes hydrogen peroxide from chloroplasts and cells (Li et al., 2013; Cao et al., 2015). CAT forms H_2_O and O_2_ in peroxisomes and ethidiomes by directly decomposing hydrogen peroxide (Mittler, 2006). The antioxidant enzyme activities (SOD, POD, and CAT) of woad leaves were increased by mild, moderate, and severe water deficits. This result may be the response of woad plants to eliminate the ROS produced by water deficits and to adapt to the stressful environment (Ghanbari and Sayyari, 2018). This response was consistent with previous studies (Sun et al., 2013; Liu et al., 2017). The antioxidant enzyme activity of Dendrobium moniliforme was increased by drought stress and rehydration (Wu et al., 2016). The activities of SOD, POD and CAT in the vegetative and fleshy root growth of woad leaves under water deficit were higher than those in the seedling stage. This result indicates that the antioxidant enzyme activities of woad leaves increased with plant growth under water stress. This was consistent with the results of Hao et al. (2019). The SOD, POD, and CAT activities of woad leaves were weakened when entering the fleshy root maturity stage. This result might have happened due to the senescence of woad plants at the end of the growth stage when the physiological activities were weakened, and the plants were less able to adapt to external stresses. The variation trends of SOD, POD and CAT in woad leaves under water stress were similar, but the increased rate of enzyme activity and the peak period of enzyme activity differed. This demonstrates their synergistic effect on avoiding oxidative damage in plants. Different antioxidant enzyme activities were correlated with Pn by linear fitting. Significant negative linear correlations existed between SOD activity and Pn, POD activity and Pn, and CAT activity and Pn at three different growth stages.

Water deficit irrigation facilitates mechanisms that improve crop drought tolerance, affecting dry matter formation and distribution (Bloch et al., 2006). Deficit irrigation effectively improves the WUE of crops compared to conventional irrigation (Wei et al., 2016). In this study, the results indicated that WUE could be improved by mild water deficit in vegetative and fleshy root growth without reducing yield. In contrast, the yield and WUE were not improved by moderate or severe water deficits. This was comparable to the findings obtained by Li et al. (2019). They showed that the dry matter of primary roots was slightly increased by a mild water deficit and that the yield was severely decreased by severe deficit treatments. The total yield and fruit number of watermelon plants are decreased by severe water deficits (Abdelkhalik et al., 2019). In our results, the yield and WUE were significantly lower in plants in T6 compared to plants in T4. This result indicates that the yield and WUE of woad were also related to the water deficit period. Crops also respond differently to water deficits in various growth periods (Santos et al., 2019). There was a close relationship between photosynthetic parameters and crop yield (Liu et al., 2016). This was similar to our results, where a highly significant positive correlation was found between the Pn and the yield of woad, and a significant positive correlation was found between the Pn and WUE.

## 5 Conclusion

The response mechanism of woad to water deficit was investigated by analyzing the changes in photosynthetic parameters, malondialdehyde, osmoregulatory, antioxidant enzyme activities, and yield. The net photosynthetic rate (Pn), transpiration rate, and stomatal conductance of woad leaves were negatively affected by moderate and severe water deficits. Still, the values of leaf water use efficiency were high with moderate and severe water deficits. The concentration of malondialdehyde gradually increased with increasing water stress in woad plants. Under water deficits, the osmotic regulator (proline) and antioxidant enzyme activities of woad were increased to repair oxidative damage. The drought tolerance of the plants was improved by increasing the proline concentration. However, the yield of woad was significantly decreased by moderate and severe water deficits. Nevertheless, the responses to physiological indicators induced by mild and moderate water deficits were reversible and alleviated upon rehydration. A significant negative correlation was found between antioxidant enzyme activity and Pn, and a linear equation was fitted. The yield of woad was not significantly reduced in the continuous mild water deficit compared to the control. Still, water use efficiency significantly increased compared to the control. Therefore, persistent mild water deficit during vegetative and fleshy root growth is recommended as the optimal irrigation strategy for woad production in cold and arid Chinese northwestern regions. The results of this study have important implications for the cultivation and sustainable development of woad.

## Data availability statement

The original contributions presented in the study are included in the article/supplementary material. Further inquiries can be directed to the corresponding author.

## Author contributions

CZ prepared the experimental scheme, data analysis and drafted the article. HZ was responsible for the funding acquisition. HZ and SY revised the experimental protocol and article format. CZ and FL performed part of the experiments and provided some of the experimental results for the manuscript. All authors contributed to the article and approved the submitted version.
